# Mental association of time and valence

**DOI:** 10.3758/s13421-023-01473-9

**Published:** 2023-10-16

**Authors:** Rolf Ulrich, Irmgard de la Vega, Verena Eikmeier, Fritz Günther, Barbara Kaup

**Affiliations:** 1 Department of Psychology, Faculty of Science, University of Tübingen, Schleichstr. 4, 72076 Tübingen, Germany; 2 Department of Psychology, Humboldt University at Berlin, Berlin, Germany

**Keywords:** Concepts, Time perception, Valence

## Abstract

Five experiments investigated the association between time and valence. In the first experiment, participants classified temporal expressions (e.g., past, future) and positively or negatively connotated words (e.g., glorious, nasty) based on temporal reference or valence. They responded slower and made more errors in the mismatched condition (positive/past mapped to one hand, negative/future to the other) compared with the matched condition (positive/future to one hand, negative/past to the other hand). Experiment 2 confirmed the generalization of the match effect to nonspatial responses, while Experiment 3 found no reversal of this effect for left-handers. Overall, the results of the three experiments indicate a robust match effect, associating the past with negative valence and the future with positive valence. Experiment 4 involved rating the valence of time-related words, showing higher ratings for future-related words. Additionally, Experiment 5 employed latent semantic analysis and revealed that linguistic experiences are unlikely to be the source of this time–valence association. An interactive activation model offers a quantitative explanation of the match effect, potentially arising from a favorable perception of the future over the past.

**Supplementary Information:**

The online version contains supplementary material available at 10.3758/s13421-023-01473-9.

The unique cognitive capacity of humans allows them to navigate mentally through time, moving between memories of the past and anticipations of the future. In this process, different time frames are imbued with significance, potentially leading to an association between time and valence. For instance, the future may be perceived as uncertain, evoking negative feelings due to the aversive nature of uncertainty (e.g., Carleton, 2016; Vazard, 2022). On the other hand, despite its uncertainty, individuals may hope for positive outcomes in the future, leading to a more positive perception of the future than the past and may thus represent an optimistic bias (e.g., Sharot et al., 2011; Weinstein, 1980). However, as explained below, the association between valence and time could also arise from subtle cognitive factors, such as how time and valence are mentally represented (i.e., de la Vega et al., 2012; von Sobbe et al., 2019). Alternatively, language experience could play a role, shaping specific associations between time and valence in people’s minds. While all four views suggest a mental link between time and valence, they propose different directions for this potential linkage. This study aims to investigate this potential connection through implicit cognitive approaches, mainly employing reaction time (RT) to shed light on the matter.

Usually, studies use questionnaires with rating scales to examine potential associations between time and valence (e.g., Berntsen & Bohn, 2010; D’Argembeau & Van der Linden, 2004; Newby-Cark & Ross, 2003; Rasmussen & Berntsen, 2013). The results of these studies are somewhat mixed. In one study, participants recalled and forecasted personally essential events (Newby-Cark & Ross, 2003). The anticipated events were usually rated more positively than the remembered counterparts. In a further experiment, positive future events were generated faster than negative future events, although past events’ recall speed was independent of their valence. These findings suggest that future negative events are more difficult to imagine than future positive events, supporting the idea that future time and positive valence are associated. Nevertheless, a study by Rubin (2014) has challenged the generality of these findings, because participants rated troubling future events as more negative than troubling past events. The context of recall in this study, however, was restricted to specific categories of events that involved traumatic events, which may let the future appear more troubling than the past. Nevertheless, under less restricted situations, people tend to view the future as more positive than the past (however, see Walker et al., 2003).

Recently Kaup et al. (2021) probed the mental association between time and valence with a less direct approach—that is, with a sentence completion task. Participants were presented with an initial sentence fragment and asked to complete the sentence by choosing one of two possible completion phrases. Participants tended to select a phrase referring to the future when the initial fragment contained a positive event and a phrase referring to the past when the initial fragment contained a negative event. This result suggests a mental link between valence and time, potentially because people view the future as positive.

As mentioned above, the mental link between time and valence may emerge or at least be additionally reinforced by subtle cognitive factors that operate outside conscious awareness. For example, in several RT studies, participants judged the tense (past vs. future) of words or sentences (cf. von Sobbe et al., 2019). Shorter RTs were observed when they were asked to respond with the left hand to the past and the right hand to the future compared with a reversed stimulus–response (S–R) mapping (e.g., Santiago et al., 2007; Ulrich & Maienborn, 2010; Weger & Pratt, 2008). This space-time congruency effect is usually attributed to a mental time line that runs from the left to the right in Western cultures (see Eikmeier et al., 2016). Furthermore, other studies have demonstrated a mental association between space and emotional valence (e.g., Casasanto, 2009; de la Vega et al., 2012). For example, Casasanto (2009) asked participants to sort good and bad items on the right and left sides in front of them. Right-handers tended to sort good items to the right and bad items to the left; the effect was reversed for left-handers. Moreover, de la Vega al. (2012) used a speeded valence-judgment task to categorize positive versus negative valenced words. Right-handers responded faster with the right hand to positive words and with the left hand to negative words compared with a reversed S–R mapping. For left-handers this effect was reversed. This space–valence association has been attributed to motor fluency of one’s dominant hand (Casasanto, 2009; de la Vega et al., 2013). Regardless of the precise interpretation of this association, lateral space appears to play an essential role in the cognitive representation of emotional valence, just as space does in time. This similarity of these associations suggests that valence (negative/positive) and deictic time (past/future) may be linked because they are mediated by spatial thinking.

The primary objective of this study is to comprehensively examine the potential mental association between time and valence. To achieve this goal, we will build upon the findings from RT research reviewed in the preceding paragraph. The RT experiments provide strong evidence for a linkage between time and valence, in which the future is associated with positive valence and the past with negative valence. This conclusion is reinforced in a fourth experiment, in which participants rated future-related words more positively than past-related words. Finally, the last experiment assesses whether even language experience could shape this mental linkage between time and valence.

## Experiment 1

Table 1 shows the design of this experiment. In each trial, right-handed participants made a speeded categorization in response to a single target word, either a valence word or a time word. In the case of valence words, the categorization was between positive and negative words, and in the case of time words, the categorization was between past and future words. Responses were given through left and right key presses. For each domain (valence or time), there were two different S–R assignments (past-left vs. past-right; negative-left vs. negative-right), and these two assignments were factorially manipulated. As a result, the two assignments either *matched* according to the association proposed above (i.e., past and negative were assigned to one response and future and positive to the alternative response) or *mismatched* (i.e., past and positive were assigned to one response and future and negative to the alternative response). Assuming that both domains are associated, we expected for this sample of right-handed participants shorter RTs in the match condition than in the mismatch condition.

**Table 1:** Design of Experiment 1

S–R assignment		Experimental factors		
**Left response**	**Right response**	**Domain**	**Congruency**	**Match**
Past	Future	Time	Congruent	MATCH
Negative	Positive	Valence	Congruent	
Future	Past	Time	Incongruent	
Positive	Negative	Valence	Incongruent	
Past	Future	Time	Congruent	MISMATCH
Positive	Negative	Valence	Incongruent	
Future	Past	Time	Incongruent	
Negative	Positive	Valence	Congruent	

The first two columns show the S–R assignment. There are four types of blocks, and each block is defined by the mapping of time and valence to the left and right response. The mapping of each domain can be congruent or incongruent. Matched blocks are given when the S–R assignment of both domains is either congruent or incongruent, whereas, in mismatched blocks, only one S–R assignment is congruent

The S–R mapping could be *congruent* or *incongruent* with respect to well-documented stimulus–response mappings between valence and space on the one hand (de la Vega et al., 2012) and time and space on the other hand (von Sobbe et al., 2019). For example, if past and future were mapped to the left and right hand, respectively, this mapping was congruent for the domain time. Likewise, if negative and positive were mapped to the left and right hand, this was a congruent mapping for the domain valence, as all our participants were right-handed. We expected to replicate the classical congruency effects for time and space which would demonstrate the validity of this experimental approach. Thus this experiment factorially combined the factors *Domain* (time vs. valence), *Match* (match vs. mismatch), and *Congruency* (congruent vs. incongruent).^1^ Note that the present paradigm was akin to the implicit-association test (Greenwald et al., 1998), commonly used to assess implicit stereotypes.

### Method

#### Participants

We aimed at 80 participants—that is, 40 for each match condition.^2^ However, due to our inclusion criteria, we tested 91 participants. Seven participants were replaced due to an error rate of 20% or higher or because they had previously taken part in a similar experiment.^3^ Because of a procedure mistake, we had 44 valid participants in the match condition. Thus, we eliminated the four participants’ data sets with the highest error rates in this condition, yielding 40 participants for the match condition and 40 for the unmatched condition. Finally, we eliminated one left-handed participant such that all of the 79 participants (62 females and 17 males) were right-handed (score of ≥40 in the Edinburgh Handedness Inventory; Oldfield, 1971). All participants were native German speakers recruited from our campus, and their mean age was *M* = 24.8 years (*SD* = 5.81).

#### Stimuli

The stimulus set comprised 60 German words, mainly consisting of single adverbs or adjectives. Thirty words had a meaning referring to time, and 30 had a meaning with a positive or negative emotional valence. The time words were the same as in Eikmeier et al. (2015). Half of the time words referred to the past (e.g., *gestern* [*yesterday*]), and half of the time words referred to the future (e.g., *morgen* [*tomorrow*]). Analogously, half of the valence words had a positive meaning (e.g., *wunderbar* [*wonderful*]), and half of them had a negative meaning (e.g., *widerlich* [*disgusting*]). Twelve additional words (three of each type: past, future, positive, and negative) were used for practice trials. A complete list of the stimuli can be found in Appendix 1.

#### Apparatus and procedure

The experiment was run in sound-proofed cabins. Stimuli were presented in the center of a screen in black font against a white background. A positive, negative, future, or past word was presented in each trial. Participants were informed that they would see either a time word or a valence word and that their task was to decide whether the word referred to past or future (time words) or had a positive or negative meaning (valence words) by pressing a left or right key. The Q and 9 keys on a standard QWERTZ keyboard were used as left and right response keys.

Each trial began with a fixation cross displayed in the middle of the screen for 200 ms, followed by an empty screen for 500 ms. Next, the stimulus word appeared in the center of the screen and remained visible until the participant responded or for a maximum of 2,500 ms if no response was recorded. Following the word presentation, the screen was empty for 1,000 ms. During practice trials, feedback was displayed on the screen for 1,500 ms, indicating whether the participant’s response was correct, incorrect, or too slow. The subsequent trial began after a blank screen was shown for 500 ms. After completing the practice part, a blank screen was shown for 1,500 ms instead of feedback, and the subsequent trial started immediately after the blank screen.

In both match conditions, each word was presented once per block. Within a block, words were presented in random order. Each block started with twelve practice trials which did not enter the final data analysis. Half of the participants started with the congruent block in the match condition and continued with the incongruent block. The two blocks’ order was reversed for the other half of the participants. Half of the participants started with the time-congruent block in the mismatch condition and continued with the valence-congruent block. Again, the order of the two blocks was reversed for the other half of the participants.

As explained above, the design of the experiment yielded the between-subjects factor *Match* (match vs. mismatch) and the within-subjects factors *Congruency* (congruent vs. incongruent) and *Domain* (valence words vs. time words). We used the *lme4* package (Bates et al., 2015) for R (R Core Team, 2022, Version 4.2.1) to perform a linear mixed-effect analysis for the influence of *Match*, *Congruency*, and *Domain* on response time (RT) and the percentage of correct responses (PC). *Match*, *Congruency*, and *Domain* were considered fixed effects, whereas participants and items were entered as random intercepts. The fixed effects were tested for significance with a likelihood ratio test of the full model against the reduced model without the effect in question. We used the procedure “allFit” of the R package *afex* (Singmann et al., 2022) to check the convergence of the fit with a range of optimizers. We also performed a traditional *minF’* analysis (Clark, 1973). Because the two statistical approaches yielded very similar results, we only report the linear mixed effect analysis results.

### Results and discussion

**Fig. 1:**
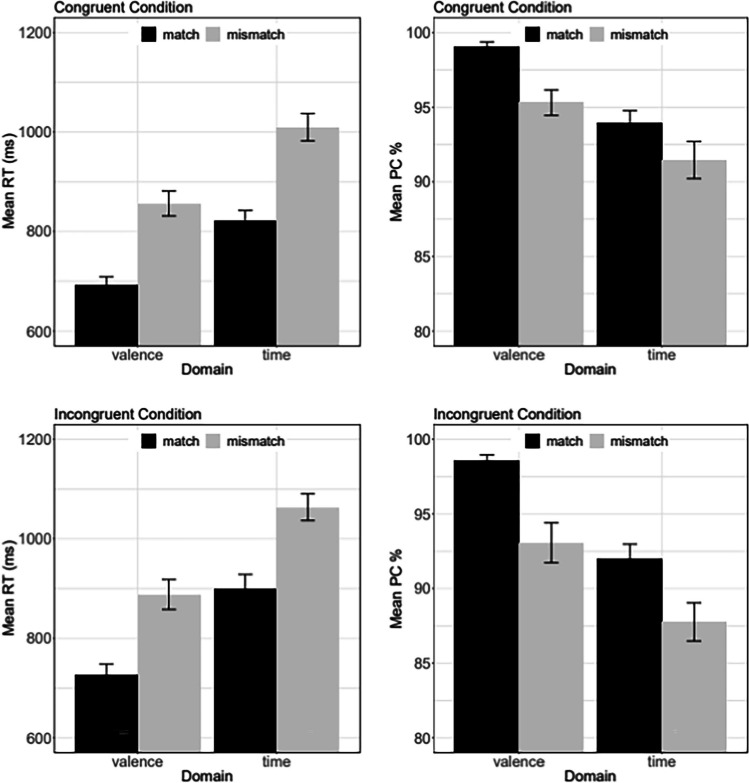
Mean reaction time (RT) and mean percentage correct (PC) in Experiment 1 as a function of Match (match vs. mismatch), Domain (valence vs. time), and Congruency (congruent condition vs. incongruent condition). The panels on the left depict the result for RT, whereas the panels on the right depict the PC result. The two top panels show the results of the congruent condition and the two bottom panels for incongruent condition. The error bars represent ±1 *SE*, where *SE* denotes the standard error of the mean. *SE* was computed with the R routine summarySE (Hope, 2022)

Trials with RTs shorter than 250 ms (0.78%) and larger than 2,000 ms (1.55%) were considered outliers and discarded from further analysis.^4^ After eliminating these trials, the overall mean PC was 93.86%. For RT analysis, only trials with correct responses were considered. Figure 1 depicts mean RT and mean PC as a function of all six experimental conditions.

As expected and theoretically most important, RTs were notably shorter in the match condition (785 ms) compared with the mismatch condition (955 ms), χ^2^ = 24.99, *p* < .001.^5^ This main effect of factor *Match* suggests that the S–R mapping applied for valence and time is not processed independently. Instead, it demonstrates that the task is performed faster when the S–R mappings of time and valence match than when they do not.

Besides this outcome, the congruent S–R mapping yielded shorter RTs than the incongruent mapping (847 ms vs. 895 ms), χ^2^ = 22.63, *p* < .001. This outcome replicates the usually reported congruency effect for time (von Sobbe et al., 2019) and valence (e.g., de la Vega et al., 2012, right-handers), which have reported congruency effects in similar millisecond ranges.

The significant main effect of *Domain* revealed that valence words were responded to more quickly than to time words (792 ms vs. 950 ms), χ^2^ = 33.12, *p* < .001. This effect presumably suggests that the processing of valence words is cognitively less demanding than the processing time words. None of the four possible interactions in this design yielded a significant effect, *p*s > .105.

Responses were more accurate in the matched condition than in the mismatched one (95.88 % vs. 91.89 %), χ^2^ = 42.33, *p* < .001. Accordingly, the observed effect of factor *Match* on RT does not reflect a trade-off between speed and accuracy but rather reflects a genuine match effect of valence and time. Moreover, response accuracy was significantly higher on congruent than incongruent trials (94.92% vs. 92.80%), χ^2^ = 3.89, *p* = .049, and also higher for valence words than for time words (96.47% vs. 91.26%), χ^2^ = 21.30, *p* < .001. The significant *Match* × *Domain* interaction, χ^2^ = 5.34, *p* = .021, revealed that the domain effect on PC was somewhat smaller for time words than for valence words. None of the three remaining interactions produced a significant effect, *p*s > .538.

In sum, the strong match effect supports the notion that the two domains are associated. Moreover, Experiment 1 replicated the classical space–time congruency effect and the space–valence congruency effect on RT. The factor *Congruency* did not significantly modulate the size of the observed match effect. The Bayes factors were BF_01_ = 13.36 and BF_01_ = 10.26 for the interactions *Match × Congruency* and *Match* × *Congruency* × *Domain*, respectively, providing strong evidence for the null hypothesis.^6^ According to the additive-factor method (Sternberg, 1969, 2001, 2011), this outcome suggests that *Congruency* and *Match* influence RT at different processing levels.

## Experiment 2

The task in Experiment 1 was spatial because left-hand and right-hand responses were employed. Thus, we wondered whether the match effect in Experiment 1 could also be observed in a nonspatial task. Experiment 2 addressed this question. Two vocal response alternatives now replaced the two spatial response alternatives. Therefore, this experiment examined whether the match effect observed in Experiment 1 also emerges when neither valence nor time is explicitly mapped to spatial response alternatives (i.e., when the RT task is nonspatial).

### Method

#### Participants

We aimed at 40 participants because we now treated *Match* as a within-subject factor. However, due to our inclusion criteria, we tested 50 participants. Nine participants were replaced because they committed too many errors (i.e., PC < 80%), and one participant because of unclear handedness (score <40 on the handedness questionnaire). The final sample consisted of 34 female and six male participants (mean age *M* = 21.9 years, *SD* = 3.49). All of them were right-handed and native speakers of German.

#### Apparatus and stimuli

The same stimuli were used as in the previous experiments. As this experiment featured vocal instead of manual responses, a microphone attached to a voice key was used instead of response keys. The voice key registered the onset of vocal responses and was used to measure RTs. The response was registered online by the experimenter. The experimenter listened to the participants’ responses via headphones outside the experimental cabin and logged the responses via key press.

#### Procedure and design

The experimental procedure was adapted to fit the vocal response requirements. The timing of single trials was changed to give participants sufficient time to respond vocally, and an extra interval in which the experimenter logged the responses was added. Specifically, as before, the stimulus word was presented for 2,500 ms or until a response was given. If no response was recorded during stimulus presentation, an empty screen was shown for another 1,500 ms or until a vocal response was detected. Consequently, participants had a maximum of 4,000 ms to respond. If no response was detected, participants received written feedback on the screen for 2,000 ms that they responded too slowly. An empty screen followed this for 2,000 ms (1,800 ms in practice trials). During this time, the experimenter logged the vocal response. In practice trials, the participants received feedback (“correct”/“wrong”) on the screen for 2,000 ms. The subsequent trial started after 1,500 ms (500 ms in practice trials).

There were two different conditions in this experiment. In one condition, the S–R mapping for valence and time words matched; that is, participants gave one response (e.g., “ke”) to future words and positive words and another response (e.g., “ko”) to past and negative words. In the other condition, the S–R mapping for valence and time words mismatched. That is, participants responded with the same syllable (e.g., “ta”) to future and negative words and with another syllable (e.g., “ti”) to past and positive words. In addition, the vocal response sets (e.g., “ko” and “ke” vs. “ta” and “ti”) changed between blocks to avoid carryover effects.

The experiment consisted of four blocks (two blocks of each match condition) arranged to counterbalance practice effects. Conditions were alternated blockwise throughout the experiment, and half of the participants started with the match condition, and half started with the mismatch condition. Each block started with 12 practice trials followed by 60 experimental trials. Within a block, words were presented in random order, and each word was presented once in each block. Response sets were counterbalanced across participants and conditions. The experiment yielded a 2 × 2 within-subjects design, with factors *Match* (match vs. mismatch) and *Domain* (time vs. valence).

### Results and discussion

**Fig. 2:**
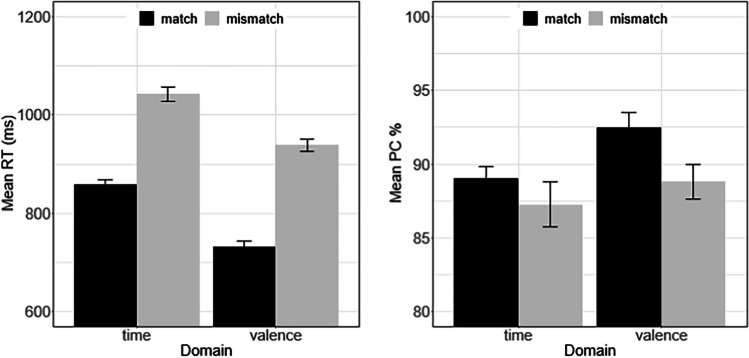
Mean response time (RT) and mean percentage of correct (PC) in Experiment 2 as a function of stimulus domain (time vs. valence) and time–valence match. The error bars reflect ±1 *SE*, where *SE* denotes the within-subject error of mean computed according to Cousineau (2007) with the correction suggested by Morey (2008). *SE* was computed with the R routine summarySE (Hope, 2022)

RTs shorter than 250 ms (0.96%) and larger than 2,000 ms (1.33%) were again considered outliers and discarded from further analysis. Mean PC (89.47%) was similar to the accuracy level observed in Experiment 1. Figure 2 summarizes mean RT and mean PC for all experimental conditions.

As in Experiment 1 and of major interest, RT was again shorter in the match condition (793 ms) than in the mismatch condition (983 ms), χ^2^ = 76.13, *p* < .001. Participants responded again faster to valence words (832 ms) than to time words (941 ms), χ^2^ = 29.81, *p* < .001. Also, consistent with the previous experiment, there was no significant interaction between *Match* and *Domain*, χ^2^ = 2.97, *p* = .085.

Participants tended to respond more accurately when the two domains matched rather than mismatched (90.77% vs. 88.14%), χ^2^ = 2.68, *p* = .102. In addition, response accuracy was again higher for valence words than for time words (90.71% vs. 88.20%), χ^2^ = 5.89, *p* = .015. Consistent with Experiment 1, the interaction between *Match* and *Domain* was insignificant, χ^2^ = 1.37, *p* = .241. In summary, the present experimental results are concordant with Experiment 1, although vocal responses were used instead of spatial responses. Thus, the match effect observed in Experiment 1 cannot be attributed to response-specific factors.

In the previous experiments, our participants were right-handers. Because the mapping of valence to space is modulated by handedness, we were interested to see whether the same results would also apply to left-handed participants. Therefore, in Experiment 3, we tried to replicate the results of Experiment 2 with a sample of left-handers.

## Experiment 3

Previous studies have shown that the spatial effect of emotional valence is associated with the participant’s hand (e.g., Casasanto, 2009; de la Vega et al., 2012). Specifically, participants respond faster with their dominant hand to positive words and nondominant hand to negative words. This valence-hand interaction most likely reflects individual experiences of hand actions, which are different for right- and left-handers (Casasanto, 2009; de la Vega et al., 2012, 2013; Kong, 2013; Milhau et al., 2015). The additional finding that this interaction reverses when participants cross their hands in such RT studies strongly supports this conclusion because it demonstrates that the valence-hand interaction is linked to the hands instead of bodyside (de la Vega et al., 2013). However, this interaction contrasts strongly with the time–space congruency effect, which is linked to bodyside instead of the hands because the interaction between the response side and tense remains unchanged when participants cross their hands (Bottini et al., 2015; de la Vega et al., 2016; Rolke et al., 2014). Nevertheless, studies have shown convincingly that this interaction effect between response side and time depends on cultural influences such as writing direction (e.g., Fuhrman & Boroditsky, 2010; Ouellet et al., 2010). Thus, the time–space effect has been ascribed to cultural influences rather than individual differences in handedness (e.g., Casasanto, 2009; de la Vega et al., 2013; Ouellet et al., 2010). On this background, we considered it possible that replicating Experiment 2 with left-handed participants could lead to a different result pattern even when they respond vocally instead of manually.

### Method

Apparatus, stimuli, and procedure were the same as in Experiment 2, and as before, vocal instead of manual responses were requested.

#### Participants

A sample of 40 left-handed participants took part in this experiment. Six participants had to be replaced because of unclear handedness (≥40 points in the handedness questionnaire). Another 13 participants who committed more than 20% wrong responses had to be replaced. The remaining sample consisted of 12 male and 28 female participants whose mean age was *M* = 24.0 years (*SD* = 3.0). All were native speakers of German and had normal or corrected vision.

### Results and discussion

**Fig. 3:**
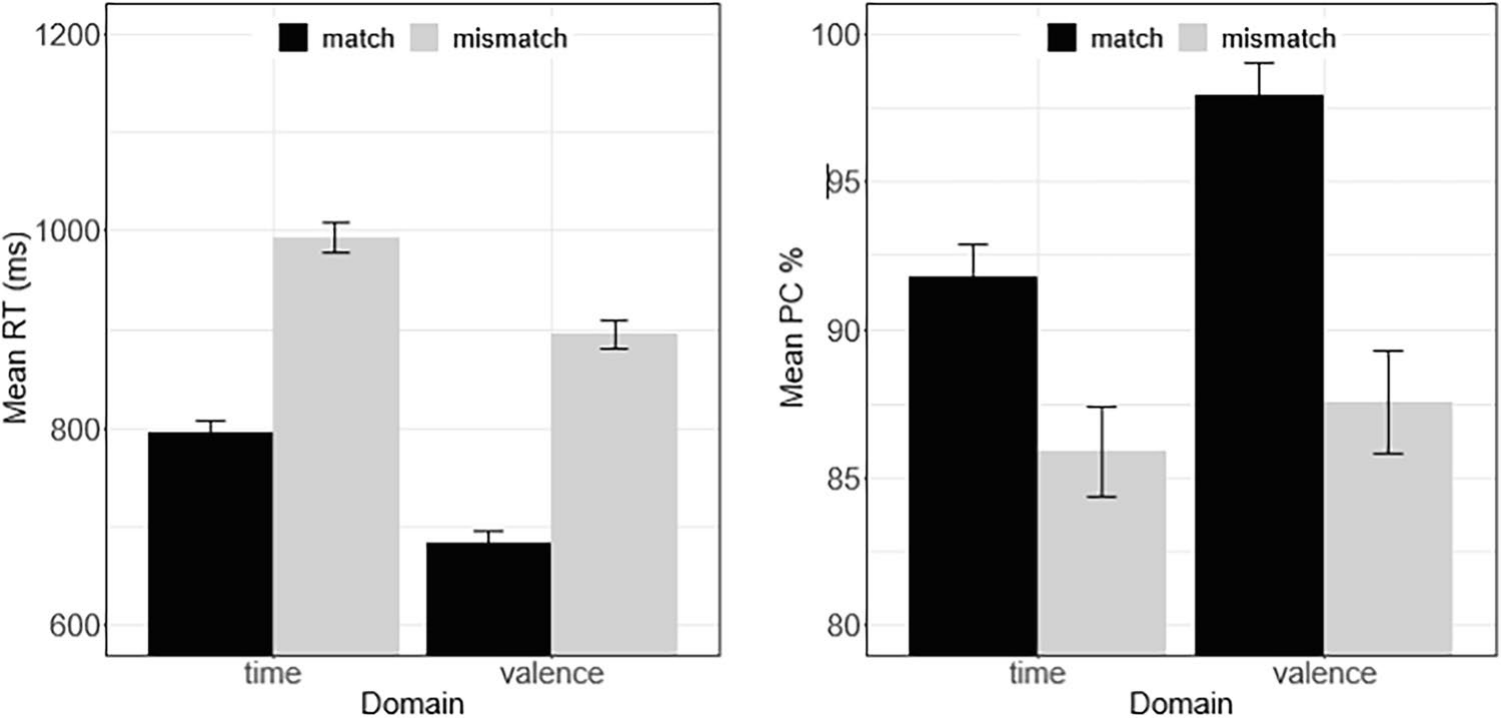
Mean response time (RT) and mean percentage of correct (PC) in Experiment 3 as a function of stimulus domain (time vs. valence) and time–valence match. The error bars reflect ±1 *SE*, where *SE* denotes the within-subject error of mean computed according to Cousineau (2007) with the correction suggested by Morey (2008). *SE* was computed with the R routine summarySEwithin (Hope, 2022)

As before, trials with RTs shorter than 250 ms (1.80%) and larger than 2,000 ms (1.05%) were removed for the main analyses. Again, the level of response accuracy (PC = 90.96%) was comparable to the previous experiments. Figure 3 summarizes the results for RT and PC. In keeping with the results of the previous two experiments and most crucially for this sample of left-handers, participants responded faster under the matched compared with the mismatched condition (739 ms vs. 932 ms), χ^2^ = 64.24, *p* < .001. Also, consistent with the previous results, responses to valence words were performed quicker than to time words (777 ms vs. 885 ms), χ^2^ = 33.93, *p* < .001, and the two factors seem to exert an additive effect on RT as before, χ^2^ = 0.62, *p* = .431. Responses were again more accurate when the two domains matched than mismatched (94.90% vs. 86.92%), χ^2^ = 53.89, *p* < .001, and also for valence words than for time words (92.96% vs. 88.93%), χ^2^ = 25.94, *p* < .001. Valence produced a larger match effect on PC than time, χ^2^ = 21.49, *p* < .001. In conclusion, Experiment 3 shows that the association between time and valence is not modulated by handedness. Thus, the results strengthen the idea that people link the future with positive valence and the past with negative valence but may be taken to question the idea that the linkage emerges from spatial thinking.

## Experiment 4

This study examines the emotional valence of time-related words utilized in Experiments 1, 2, and 3 (Appendix 1). Building upon the results of these experiments, we hypothesize that individuals tend to associate future-related words with a more positive valence than past-related words. Furthermore, we speculate that age may influence this evaluation, with younger participants potentially appraising future-related words more positively than older participants. Thus, two age groups were included in the study.

### Method

#### Participants

In the second half of April 2023, we conducted two online surveys using the software package SoSci Survey (SoSci Survey GmbH, Marianne-Brandt-Str. 29, 80807 Munich, Germany). One survey was explicitly aimed at students, while the other was targeted at employees of the University of Tübingen. The instructions for the second survey specified that participation was open to employees above the age of 50. Participation in both surveys was voluntary, and six vouchers worth 30 Euros each were raffled off as incentives. Participants were free to discontinue the survey at any point. Only fully completed questionnaires were included in the subsequent data analysis. For the purposes of this study, we classified participants below the age of 50 as “younger adults” (*N* = 826, mean age = 23.8 years, *SD* = 4.4, females = 70.9%, males = 30.0%, diverse = 2.1%), while those above the age of 50 were classified as “older adults” (*N* = 221, mean age = 58.6 years, *SD* = 6.7, females = 60.0%, males = 39.8%, diverse = 0.5%).

#### Apparatus, stimuli, and procedure

Participants rated time-related words listed in Appendix 1 using a 7-point Smiley Face Likert scale, with the two endpoints labeled as “terrible” and “excellent.” The Likert scale was presented in opposite directions to the two halves of the participants: half from left to right, and the other half from right to left. The order in which the words were presented was randomized for each participant. Each word was displayed on the computer screen above the Likert scale, and participants selected the corresponding smiley by clicking on it using the mouse pad. Once a selection was made, the screen was cleared, and the next word was presented for evaluation. The experiment lasted approximately 5 minutes.

### Results

**Fig. 4:**
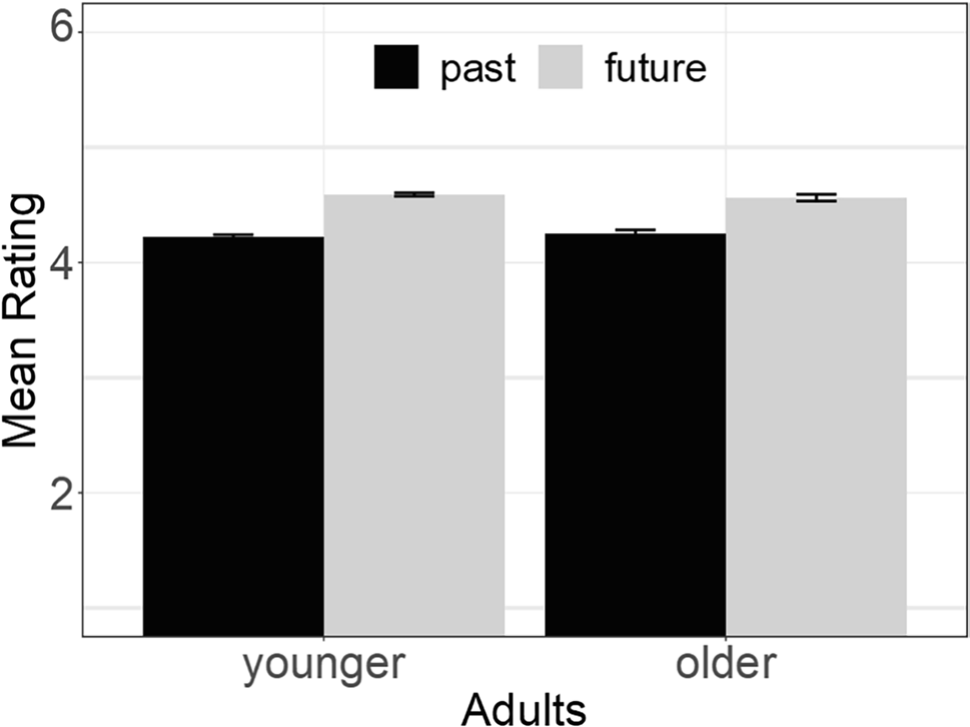
Mean rating as a function of time (past vs. future) for younger and older adults. The error bars present the 95% confidence interval. CI was computed with the R routine summarySEwithin (Hope, 2022)

For each participant, we calculated the average rating for the 15 past-related words and also the average rating for the 15 future-related words. These average ratings were then submitted to a two-way analysis of variance, with the between-subject factor Adult (younger vs. older) and the within-subject factor Time (past vs. future). Future-related words were rated more positively than past-related words, *F*(1, 4045) = 310.1, *p* < .001, *d* = 0.53, *BF*_10_ = 1.76·10^36^. However, neither the main effect of Adult, *F*(1, 1045) = 0.11, *p* = .736, *BF*_01_ = 9.11 nor the Adult × Time interaction,* F*(1, 1045) = 1.36, *p* = .244, *BF*_01_ = 5.75, were statistically significant and instead provided evidence for the null hypothesis (Fig. 4).

## Discussion

The study’s results support the notion that individuals tend to ascribe a more positive valence to future-related words than negative-related words. This finding is consistent with the present RT experiments that demonstrated a match effect, in which participants had not explicitly evaluated time-related words regarding their valence. Additionally, our results suggest that the observed valence effect in Experiment 4 is stable across different age groups, although it is important to note that our study’s older participants were still middle aged. Consequently, it is possible that the valence effect could be attenuated or even reversed in older individuals, as older adults increasingly appraise the past in a positive light (cf. Laureiro-Martinez et al., 2017). Nonetheless, this particular issue, which lies beyond the scope of our study, could be addressed in future investigations

## Experiment 5

In this final analysis, we explored language experience as a potential alternative source of the association between time and valence: For example, participants might have learned that words related to the past are more often used in negative linguistic contexts than words related to the future. In order to examine this possibility, we conducted an additional analysis using distributional semantic models (DSMs; Lenci, 2008).

DSMs track the usage patterns of words in large corpora of natural language. More specifically, they track how often words occur over pre-defined linguistic contexts (e.g., within a certain distance of other words). This distribution over contexts is represented as a high-dimensional vector. As a simple toy example, assume a case where the word TARGET appears five times in the context of WORD1, never in the context of WORD2, and three times in the context of WORD3. This would result in the distributional vector TARGET = (5, 0, 3). In a large body of literature, it has been shown that these distributional vectors serve as useful meaning representations for words (for a recent review, see Günther et al., 2019), in line with the distributional hypothesis that words with similar meanings are used in similar contexts (Lenci, 2008).

These distributional vectors thus allow us to measure the semantic similarity between words, typically defined as the cosine similarity between their distributional vectors. If two words are always used in the same contexts (i.e., perfectly interchangeable synonyms), their distributional vectors will be identical, resulting in a cosine similarity of 1; if they are never used in the same contexts, the cosine similarity between the distributional vectors will be 0. Using this approach, we investigated whether the association between time and valence would also be reflected in the cosine similarity scores between the four categories of words used in our experiments (positive, negative, past, and future).

### Method

For this analysis, we employed three different German distributional semantic models provided in the online repository by Günther et al. (2015). The first (*dewak100k_lsa*) is an LSA (Latent Semantic Analysis; Landauer & Dumais, 1997) model that constructs distributional vectors starting from word-by-document counts. Consequently, words that often occur together will end up with similar LSA vectors, and LSA tends to produce high cosine similarities for associatively related words (Jones et al., 2006). The second (*dewak100k_hal*) is a HAL (Hyperspace Analogue to Language; Lund & Burgess, 1996) model that constructs distributional vectors from word-by-word counts (as in the example above). Consequently, words surrounded by the same words and thus mutually replaceable will end up with similar HAL vectors, and HAL tends to produce high cosine similarity for semantically related words (Jones et al., 2006). The third (*dewak100k_cbow*) is a word2vec model of the cbow variant (continuous bag-of-words; Mikolov et al., 2013) is a conceptual development of HAL which employs a neural network predicting words from their surrounding context words rather than word-by-word counts. All three models were built from the same large German corpus, deWaC (Baroni et al., 2009). For further technical details on these models, see their descriptions in the repository by Günther et al. (2015).

Using these models, we used the R package *LSAfun* (Günther et al., 2015) to compute the cosine similarities between all possible word pairs consisting of a time word and a valence word used in Experiments 1–3.

### Results and discussion

**Fig. 5:**
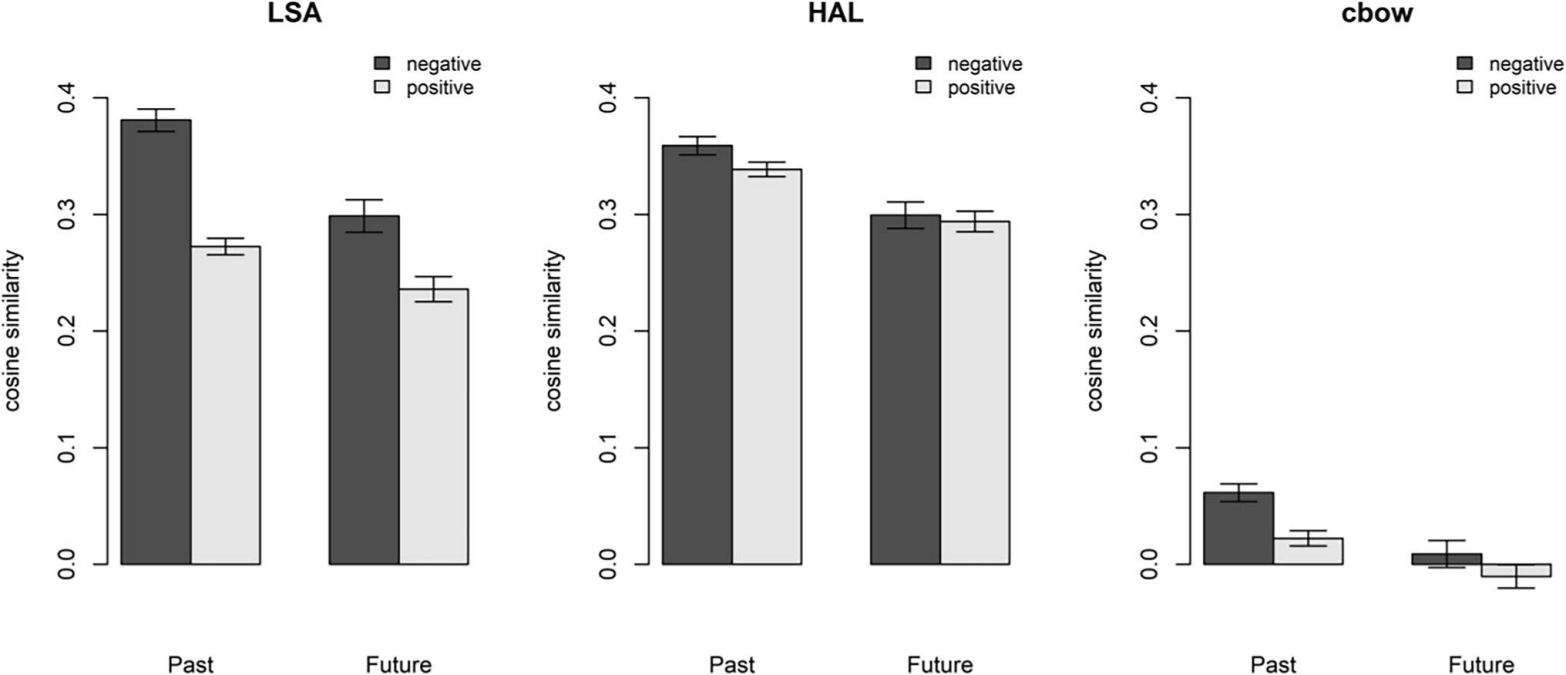
Cosine similarity scores for the four categories of words used in Experiments 1–3. The error bars reflect ±1 *SE*

The similarity patterns are depicted in Fig. 5. Analyses of variance show a main effect of time with the cosine similarities between time and valence words being higher for past than future words for all three models—LSA: *F*(1, 864) = 33.12, *p* < .001; HAL: *F*(1, 864) = 37.77, *p* < .001; cbow: *F*(1, 864) = 23.33, *p* < .001. There was also a main effect of valence with the cosine similarities between valence and time words being higher for negative compared with positive words for the LSA model, *F*(1, 864) = 77.65, *p* < .001, and the cbow model, *F*(1, 864) = 12.48, *p* < .001, but not for the HAL model, *F*(1, 864) = 2.76, *p* = .097. We also observed an interaction between time and valence, but only for the LSA model, *F*(1, 864) = 4.99, *p* = .026, where the difference between negative and positive words was larger for past words than for future words, and not for the other models—HAL: *F*(1, 864) = 0.78, *p* = .377; cbow: *F*(1, 864) = 1.28, *p* = 0.258. Thus, these analyses show little to no evidence that the association between time and valence observed in our RT experiments is also reflected in the distributional semantic similarities of the respective word categories. Linguistic experiences are, therefore, not a likely source of the observed association.

## General discussion

This study used response time (RT) as the primary measure to investigate the potential association between time and valence. The results of the study provided strong evidence of such an association but questioned the idea that this linkage can be attributed to spatial thinking. The data showed that when participants were asked to map negative words with past and positive words with future, they responded relatively faster. This effect was consistent across all three experiments, regardless of whether participants provided manual or nonspatial vocal responses, and regardless of whether they were right- or left-handed. Additionally, Experiment 4 further supported these findings by demonstrating that when participants rated the valence of time-related words directly, they rated future-related words as more favorable than past-related words, which aligned with the match effect found in the RT experiments. In conclusion, this study’s results suggest a robust mental association between time and valence. Finally, Experiment 5 indicates that this association does not emerge from linguistic experiences.

One may wonder, however, whether the RT paradigms of this study create the observed match effect—an effect that possibly did not preexist before the experiment. In order to address this possibility, we compared the match effect between the first and second half of each experiment. If the effect is introduced within an experiment, one expects that the effect will be larger for the second than the first half. Table 2 contains the outcome of this additional analysis. The results of Experiments 1 and 2 argue against the view that the effect was established during the experiment. However, in Experiment 3 with left-handed participants, the match effect is more prominent for the second than the first half. As can be seen in Table 2, mean RT benefits, especially in the match condition, from practice, whereas accuracy, especially from practice in the mismatch condition.

**Table 2:** Size of the match effect for each experiment’s first versus the second half

Experiment	First half of the experiment	Second half of the experiment	Sig. of the interaction effect
	Mismatch − Match = Size	Mismatch − Match = Size	
1	957 − 786 = 171 ms 91.2 − 95.8 = −4.6%	936 − 776 = 160 ms 92.7 − 96.0 = −3.3%	*p* = .741 *p* = .703
2	1014 − 804 = 210 ms 87.8 − 92.4 = −4.6%	972 − 789 = 183 ms 88.2 − 90.2 = −2.0%	*p* < .001 *p* = .220
3	934 − 785 = 149 ms 80.5 − 94.0 = −13.5%	930 − 724 = 206 ms 89.0 − 95.2 = −6.2%	*p* < .001 *p* < .001

The upper/lower line in each cell gives the difference in mean RT/percentage correct between the two experimental conditions. The last column shows the statistical significance of the Experimental Half × Match effect

Explaining this difference between right-handed and left-handed individuals is difficult. However, it may be due to the dominant hand being associated with positive valence (e.g., Casasanto, 2009; de la Vega et al., 2012): For left-handed individuals, it seems possible that the left side of the body is associated with positive valence and the right side with negative valence at the start of the experiment. This association between body side and valence in left-handed individuals may interfere with the experimental responses required in the match condition of future (right side of the body) and positive valence (left side of the body) requiring one response, and past (left side of the body) and negative valence (right side of the body) requiring the alternative reaction. In right-handed individuals, however, such a pre-existing linkage between body side and valence should have a beneficial effect on RT in the match condition, with past (left side of the body) and negative valence (left side of the body) requiring one response and future (right side of the body) and positive valence (right side of the body) the alternative response. In summary, the opposing modulation of the match effect with practice in left- and right-handers could have arisen because of a valence-body-side interaction between these two groups of individuals; this interaction would facilitate the match effect for right-handers but hamper it for left-handers. However, the strong match effect in Experiment 3 demonstrates that even left-handers must associate the future with positive valence and the past with negative valence.

**Fig. 6:**
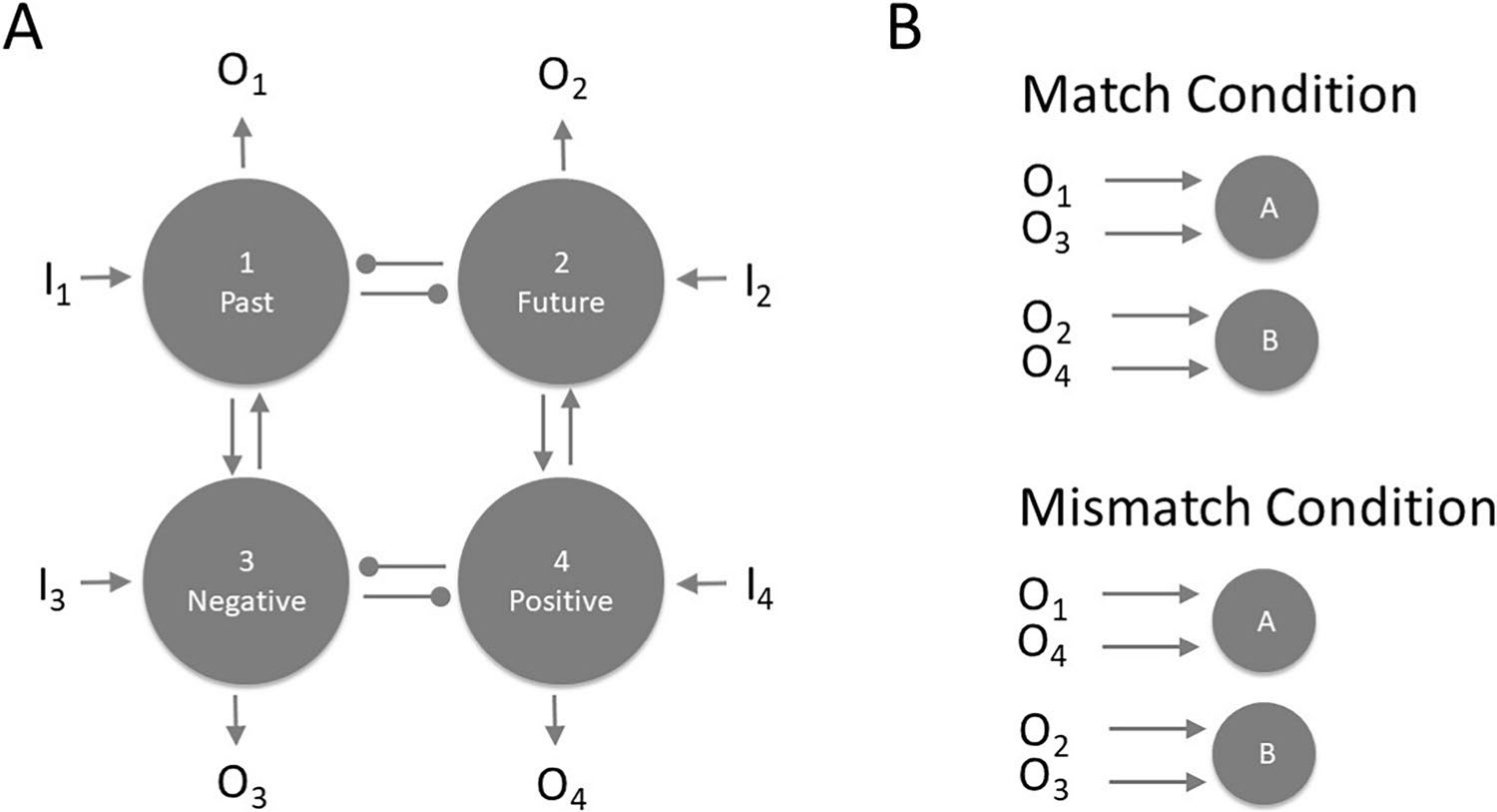
An interactive activation model to account for the match effect observed in Experiments 1–3. **A** Architecture of this model. Four nodes represent past time (1), future time (2), negative valence (3), and positive valence (4). Mutual inhibition acts between Nodes 1 and 2 and between Nodes 3 and 4. By contrast, mutual excitation acts between Nodes 1 and 3 and between Nodes 2 and 4. Stimulus input activates the corresponding node. For example, past-related information activates Node 1 via input *I*_*1*_. The concerted outputs *O*_*i*_ (i=1,...,4) of all four nodes determine the dynamic of response activation. **B** How these outputs drive the activation of the response Nodes A and B under the Match condition and the Mismatch condition.

**Fig. 7:**
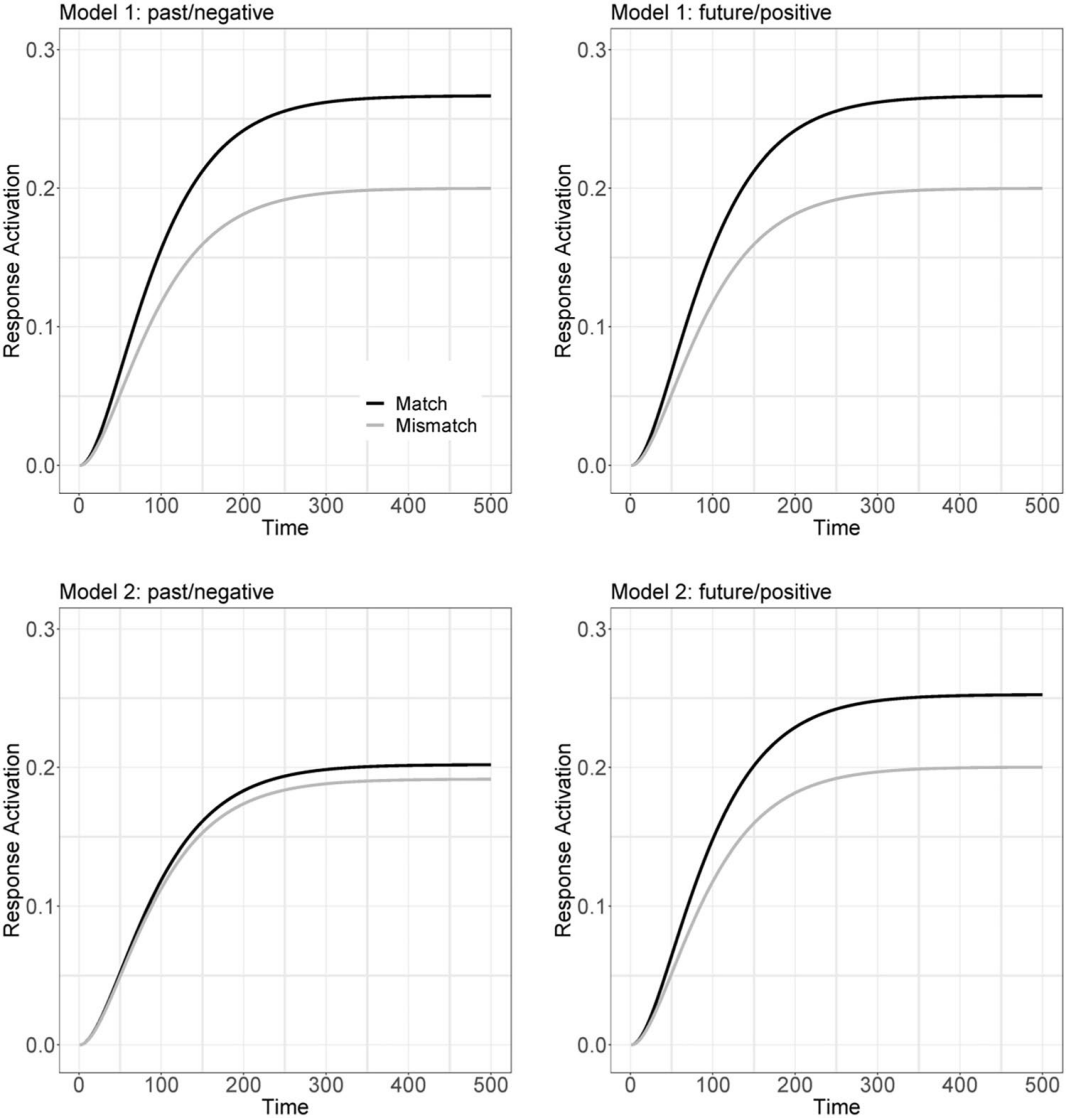
Predicted response activation as a function of time and Match condition for Model 1 (upper two panels) and Model 2 (lower two panels). The two left panels depict these functions for responses to past or negative words, whereas the panels on the right for responses to future or positive words. Appendix 2 contains the computational details

In the following, we will present a simple quantitative account of the match effect. Although the account is ad-hoc, it may help to guide further research on the observed association between time and valence. Most crucially, the model suggests the possibility of symmetrical and asymmetrical associations between time and valence, implying somewhat different predictions on RT. The account is a simple variation of the parallel distributed processing idea (cf. Rumelhart et al., 1988, Chapter 2). Two model versions are considered (Fig. 6). Model 1 assumes that the mental representations of past and future act as antagonists inhibiting each other. The same assumption applies to negative and positive valence. By contrast, past and negative, as well as future and positive, act as agonists exciting each other. In the match condition, past and negative activate the same response alternative, and future and positive activate the other response alternative. However, past and positive activate the same response alternative in the mismatch condition, and future and negative activate the other response alternative. Figure 7 (upper panels) depicts the predicted response activation as a function of time under both matching conditions (see Appendix 2 for computational details). The left upper panel reveals the model’s prediction for responses to past or negative words (past/negative responses), whereas the right upper panel depicts the predictions for future or positive words (future/negative responses). For all words, response activation develops faster and to a higher level under the match condition than under the mismatch one. Thus, Model 1 can account for the observed match effect on RT.

An alternative Model 2 also seems plausible and is also consistent with the observed match effect. Specifically, as discussed in the introduction, there is evidence from rating studies that imagined future events are more positive and idyllic than their past counterparts (e.g., Berntsen & Bohn, 2010; D’Argembeau & Van der Linden, 2004; Newby-Cark & Ross, 2003; Rasmussen & Berntsen, 2013). Therefore, one might speculate whether the association between time and valence is solely driven by future time and positive valence because linking future time and positive valence promotes a motivated perspective with one’s life, thereby serving as a functional property for human beings (Kaup et al., 2021). To evaluate the prediction of this possibility, we eliminated the exciting links between the nodes representing past time and negative valence in Model 1 but kept the exciting links between the nodes representing future and positive. Hence only future time and positive valence would still act as agonists in Model 2. In contrast to Model 1, this alternative predicts that there should only be a match effect for future/positive words but none or a highly reduced match effect for past/negative words (as shown in the lower two panels of Fig. 7).

To distinguish between the two alternative models, we reanalyzed the data from Experiments 1–3. Specifically, we computed the match effect separately for past/negative words and future/positive words for all data sets (Figs. 1, 2, and 3 in the Supplementary Material). Unfortunately, the results are somewhat mixed: Only in Experiment 1 was the match effect significantly modulated by the response category (i.e., past/negative vs. future/positive words), χ^2^ = 6.18, *p* = .013; as implied by Model 2, the effect size was larger for future/positive words (184 ms) than for past/negative words (151 ms).^7^ Thus there is some slight evidence that the match effect may originate from a particularly strong mental link between future and positive valence.

In summary, the findings of this study indicate a favorable perception of the future in comparison to the past. This inclination may be attributed to the common tendency among individuals to exhibit unrealistic optimism toward future outcomes, despite factual evidence that challenges such a perspective (e.g., Sharot et al., 2011; Weinstein, 1980). Our study suggests that this association between positive valence and time may have a robust cognitive and emotional basis, possibly due to the role of an optimistic outlook as a driving force for overall life satisfaction and well-being (e.g., Kaup et al., 2021). It is essential to acknowledge that these conclusions are confined to the specific population under investigation—namely, students and university staff at the University of Tübingen, whose life circumstances are assumed to be generally positive.

## Conclusion

The present experiments demonstrate a strong association between time and valence. Responses are faster when past and negative words are mapped to one response and future and positive words to the alternative response. Moreover, participants rated future-related words more positively than past-related words. We also examined whether or not the origin of the match effect is rooted in our linguistic experience. However, the results of the present experiments are rather inconsistent with such a view. An interactive activation account can explain this association, and our modeling suggests that the association is rooted in a strong link between future time and positive valence, which could reflect an optimistic view of the future.

### Supplementary Information

Below is the link to the electronic supplementary material.

Supplementary file1 (DOCX 530 KB)

## Data Availability

All data and codes can be found online: https://osf.io/uys6g/?view_only=003444519d014499a7b693410d31a3fa

## Appendix 1: Stimulus material

The following words were used as stimuli in the main session of Experiments 1, 2, and 3. The approximate English translation is based on the Cambridge Dictionary (online version)

**Table:**

	Past words	Future words	Negative words	Positive words
1	damals *at that time*	absehbar *predictable*	fürchterlich *awful*	bestmöglich *at its best*
2	ehemals *formerly*	anstehend *pending*	scheußlich *horrid*	hervorragend *excellent*
3	einst *long ago*	*bald* *soon*	verhasst *hated*	genial *genial*
4	einstmals *formerly*	bevorstehend *forthcoming*	gemein *mean*	wunderbar *wonderful*
5	früher *previous*	demnächst *soon*	mies *grotty*	prächtig *glorious*
6	gestern *yesterday*	in Zukunft *in future*	miserabel *lousy*	erstklassig *first-class*
7	jüngst *recently*	kommend *to come*	schlimm *bad*	einwandfrei *flawless*
8	kürzlich *recently*	künftig *in future*	widerlich *obnoxious*	grandios *terrific*
9	letztens *recently*	morgen tomorrow	hässlich *ugly*	traumhaft *dreamlike*
10	neulich *recently*	nachher *afterwards*	abscheulich *abominable*	großartig *grandiose*
11	seinerzeit *back then*	nächstens *shortly*	schrecklich *dreadfully*	paradiesisch *paradisiac*
12	unlängst *recently*	nahend *approaching*	garstig *nasty*	himmlisch *heavenly*
13	vergangen *past*	später *later*	grässlich *direful*	brilliant *brilliant*
14	vorgestern *the day before yesterday*	übermorgen *the day after **tomorrow*	böse *evil*	vortrefflich *admirable*
15	vorhin *just now*	zukünftig *in the future*	abstoßend *off-putting*	herrlich *magnificent*

## Appendix 2: Computational details of Models 1 and 2

Model 1 is depicted in Fig. 6. It assumes four nodes: Node 1 represents the past, Node 2 future, Node 3 negative valence, and Node 4 positive valence. For example, the presentation of a word referring to a past word would activate Node 1. Likewise, the presentation of a future, negative, or positive word would activate Nodes 2, 3, or 4, respectively. In the following, we will outline the computation steps for the presentation of a past word. The computational steps for the three other word cases are analogous. The presentation of a past word produces a gradual input *I*_*1*_ to Node 1, which propagates to its neighbor nodes. Specifically, Node 1 inhibits Node 2 and excites Node 3. Node 1 also excites the response Node *A* that is associated with a past word.

Let *a*_*i*_*(t)* denote the activation level of node *i* by time *t*, then the total net input, *n*_*i*_*(t),* to Node *i* (*i*=1,..,4) at time *t* is given by

$$\begin{array}{l}{n}_{1}\left(t\right)=h\cdot {a}_{2}\left(t\right)+f\cdot {a}_{3}\left(t\right)+{I}_{1}\left(t\right),\\ {n}_{2}\left(t\right)=h\cdot {a}_{1}\left(t\right)+f\cdot {a}_{4}\left(t\right),\\ \begin{array}{c} {n}_{3}\left(t\right)=f\cdot {a}_{1}\left(t\right)+h\cdot {a}_{4}\left(t\right),\\ {n}_{4}\left(t\right)=f\cdot {a}_{2}\left(t\right)+h\cdot {a}_{3}\left(t\right).\end{array}\end{array}$$

The input to the response node *A* depends on the matching condition. In the match condition, *A* receives excitatory inputs from Nodes 1 and 3, that is,

$$A\left(t\right)=f\cdot {a}_{1}\left(t\right)+f\cdot {a}_{3}\left(t\right),$$

and in the mismatch condition, excitatory inputs from Nodes 1 and 4,

$$A\left(t\right)=f\cdot {a}_{1}\left(t\right)+f\cdot {a}_{4}\left(t\right).$$

After each time step, a node’s activation becomes updated by employing an activation function* F*, that is,

$${a}_{i}\left(t+1\right)=F\left[{n}_{i}\left(t\right)\right],i=1,\dots ,4.$$

For simplicity, we used the identity function for *F,* the cumulative density function of the gamma distribution for *I*_*1*_ (shape parameter = 2, rate parameter =1/50), and assumed that excitation and inhibition are of the same absolute magnitude, that is, $f=\left|h\right|=0.3$. Additional investigations showed that these auxiliary assumptions would not change the qualitative predictions described in the main text.

## Footnotes

1 Domain and Congruency were manipulated within participants and Match between participants. If Match were also a within-subjects factor, participants had to relearn a new S–R mapping four times within a single session. Therefore, we employed Match as a between-subjects factor to make the task easier for the participants.

2 Based on our research on Time × Space Congruency effects, on Valence × Space Congruency effects, and some piloting work, we regarded the hypothized match effect as medium to large. Accordingly, the statistically power for a one-sided* t* test concerning a match effect would be 80% or larger.

3 We deemed an exclusion criterion of 20% or more errors to be rather lenient. For instance, if the criterion is set at 25%, it would imply that 50% of all responses were merely lucky guesses rather than accurate, stimulus-driven responses. The error rate was particularly high in the mismatch group, which already demonstrated that participants had problems in associating positive valence with the past and negative valence with the future.

4 Virtually the same results were obtained without discarding these outlier trials, which is also true for the subsequent experiments in this paper. Moreover, because RT distributions are skewed, a three-sigma rule for outlier exclusion is inappropriate. Therefore, we employed fixed cutoffs for all experiments (cf. Ulrich & Miller, 1994).

5 The degrees of freedom for this and subsequent chi-squared statistic is *df* = 1.

6 This additional analysis was performed with the R routine “BayesFactor” (Morey & Rouder, 2022).

7 Experiment 2: χ^2^ = 2.80, *p* = .094. Experiment 3: χ^2^ = 0.09, *p* = .952.
