# Supplementary material for: Mental association of time and valence
Source: Mem Cognit. 2023 Oct 16;52(2):444–58. doi: 10.3758/s13421-023-01473-9 (PMC10896927; doi:10.3758/s13421-023-01473-9)

Supplementary Material

*Fmin‘* is computed from $F_{1}\left( n, n_{1} \right)=f_{1}$ and $F_{2}\left( n, n_{2} \right)=f_{2}$ as following (cf. Clark, 1973):

$Fmin\left( n,j \right)=f_{1}\cdot f_{2}/(f_{1}+f_{2}$) with $j=\left( f_{1}+f_{2} \right)^{2}/\left( f_{1}^{2}/n_{2}+f_{2}^{2}/n_{1} \right)$. We rounded *j* to the

nearest integer in the following tables. For example, *Fmin‘* for the main effect of Match in Table 1 is computed as $Fmin^{'}\left( 1, 84 \right)=\left( 29.52\cdot657.74 \right)/\left( 29.52+657.74 \right)= 28.25$

with $j=\left( 29.52+657.74 \right)^{2}/\left( {29.52}^{2}/58+{657.74}^{2}/77 \right)= 83.92.$

**Table 1**

| Effect | by-participants | | by-items | | Fmin’ - analysis | |
| --- | --- | --- | --- | --- | --- | --- |
|  | *F_1_* (1, 77) | *p_1_* | *F_2_* (1, 58) | *p_2_* | *Fmin’* | *p* |
| Match | 29.52 23.30 | < .001 < .001 | 657.74 34.54 | < .001 < .001 | F(1,84) = 28.25  F(1,135) = 13.91 | <.001  <.001 |
| Congruency | 25.17  13.93 | < .001  < .001 | 65.65  21.00 | < .001  < .001 | F(1,123) = 18.19  F(1, 134) = 8.37 | <.001  .004 |
| Domain | 240.00  40.05 | < .001  < .001 | 47.46  14.47 | < .001  < .001 | F(1,81) = 39.62  F(1,98) = 10.63 | <.001  .002 |
| Congruency × Domain | 2.82  2.11 | .097  .150 | 7.52  2.20 | .008  .143 | F(1,123) = 2.05  F(1,133) = 1.08 | .151  .301 |
| Match × Domain | 0.41  0.60 | .522  .441 | 1.21  0.72 | .277  .398 | F(1,120) = 0.31  F(1,135) = 0.23 | .581  .568 |
| Match × Congruency | 0.41  2.27 | .526  .136 | 1.31  2.70 | .258  .106 | F(1,117) = 0.31  F(1,135) = 1.23 | .577  .269 |
| Match × Domain × Congruency | 0.32  0.00 | .576  .993 | 0.99  0.03 | .325  .875 | F(1,118) = 0.24  F(1,77) = 0 .00 | .624  1.000 |

Results of Experiment 1: RT Analyses (black numbers); PC Analyses (green numbers).

**Table 2**

Results of Experiment 2: RT Analyses (black numbers); PC Analyses (green numbers).

| Effect | by-participants | | by-items | | Fmin’ - analysis | |
| --- | --- | --- | --- | --- | --- | --- |
|  | *F_1_* (1, 39) | *p_1_* | *F_2_* (1, 58) | *p_2_* | *Fmin’* | *p* |
| Match | 169.42 4.82 | <.001 .034 | 1048.60 21.00 | <.001 < .001 | F(1,52) = 145.85  F(1,57) = 3.92 | <.001  .053 |
| Domain | 96.25  5.49 | <.001  .024 | 40.46  4.14 | <.001  .046 | F(1,93)=28.49  F(1, 97) = 2.36 | <.001  .128 |
| Match x Domain | 1.61  0.67 | .211  .417 | 6.44  2.92 | .014  .093 | F(1,58) = 1.29  F(1, 57) = 0.54 | .261  .463 |

**Table 3**

Results of Experiment 3: RT Analyses (black numbers); PC Analyses (green numbers).

| Effect | by-participants | | by-items | | Fmin’ - analysis | |
| --- | --- | --- | --- | --- | --- | --- |
|  | *F_1_* (1, 39) | *p_1_* | *F_2_* (1, 58) | *p_2_* | *Fmin’* | *p* |
| Match | 131.17 28.42 | <.001 <.001 | 925.62 155.73 | <.001 < .001 | F(1,50)=114.89  F(1,53) = 24.03 | <..001  <.001 |
| Domain | 71.01  6.87 | <.001  .012 | 46.49  10.56 | <.001  .002 | F(1,97)=71.01  F(1,83) = 4.16 | <.001  .045 |
| Match x Domain | 0.78  4.41 | .211  .042 | 2.28  11.00 | .004  .002 | F(1,65)=0.58  F(1,69) = 3.15 | .449  .080 |

Figure 1. Reanalysis of Experiment 1. Mean reaction time (RT) and mean percent correct (PC) as a function of Time-Valence Match (Match vs. mismatch), Category (past/negative vs. future/positive), and Congruency (congruent condition vs. incongruent condition). The error bars represent ± 1 SE.


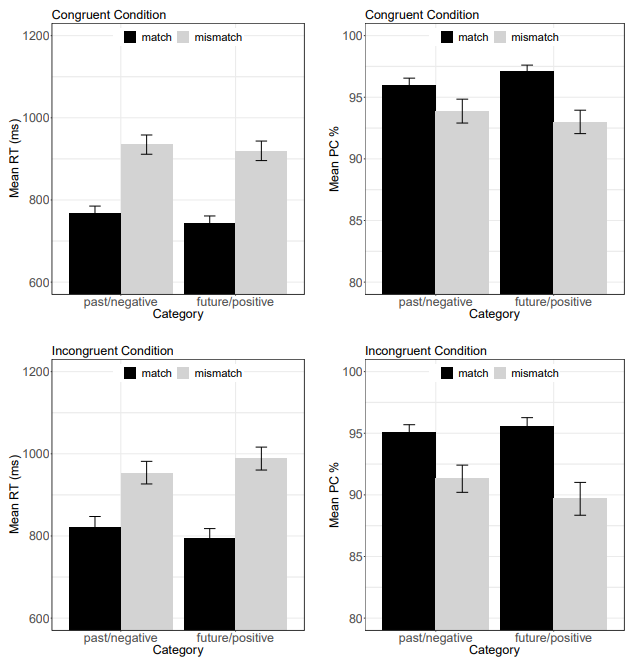


Figure 2. Reanalysis of Experiment 2. Mean response time (RT) and mean percentage of correct (PC) in Experiment 2 as a function of Category (past/negative vs. future/positive) and Time-Valence Match (Match vs. mismatch). The error bars reflect ± 1 SE.


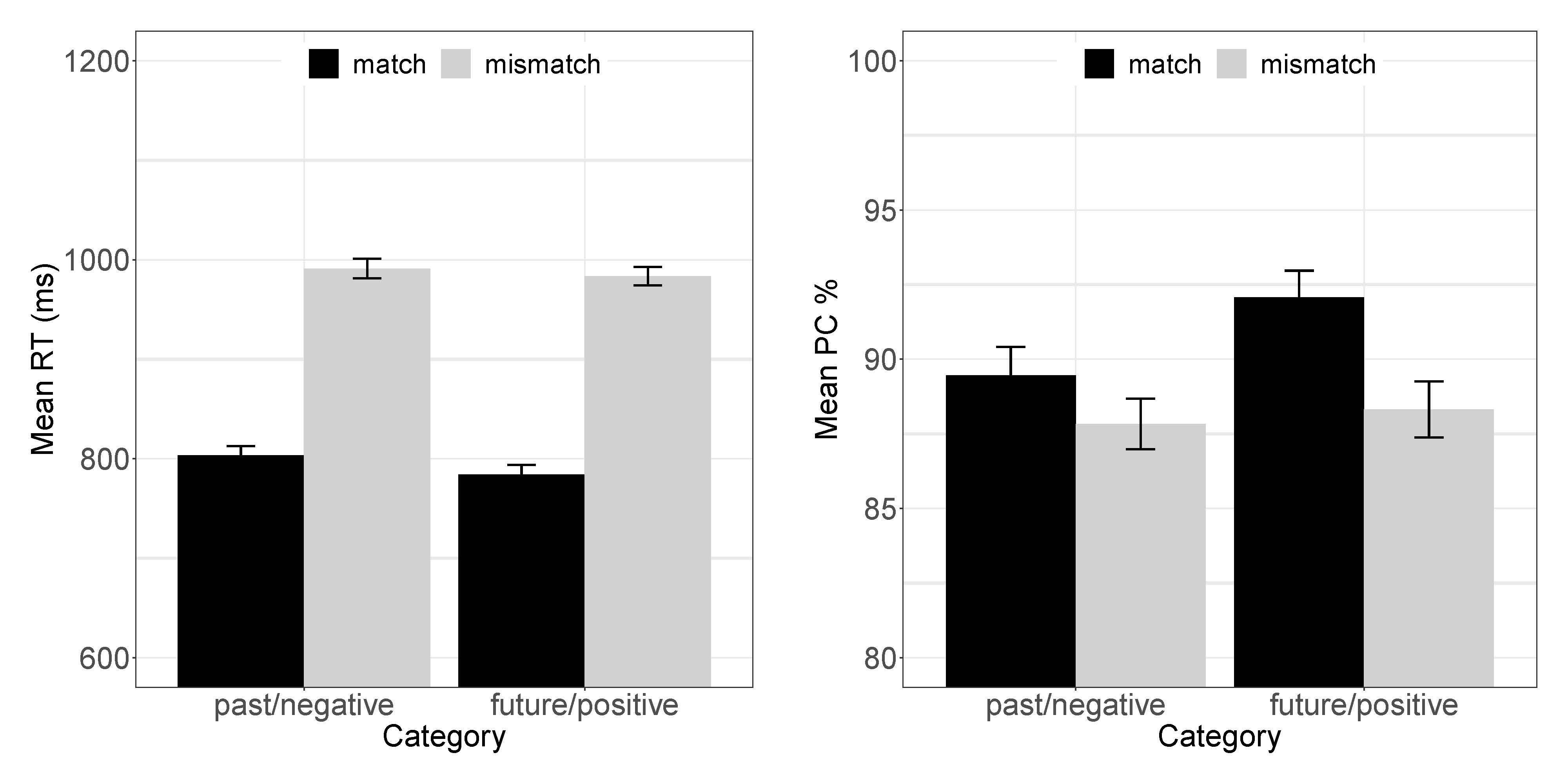


Figure 3. Reanalysis of Experiment 3. Mean response time (RT) and mean percentage of correct (PC) in Experiment 2 as a function of Category (past/negative vs. future/positive) and Time-Valence Match (Match vs. mismatch). The error bars reflect ± 1 SE.


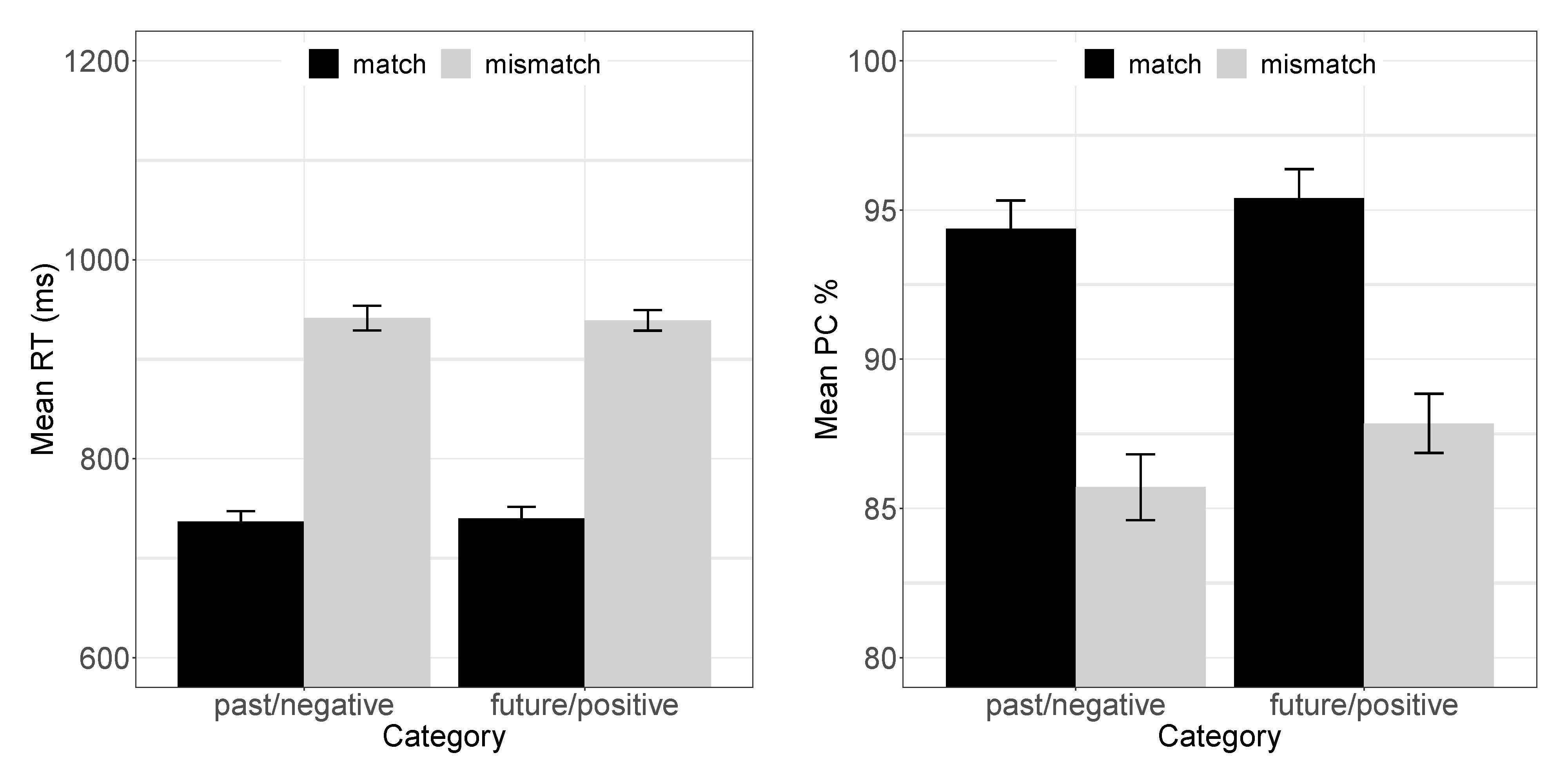

Supplement: Supplementary file 1 — Supplementary file1 (DOCX 530 KB) [file 13421_2023_1473_MOESM1_ESM.docx]
